# Supplementary material for: Comparative efficacy of treatments for previously treated patients with advanced esophageal and esophagogastric junction cancer: A network meta-analysis
Source: PLoS One. 2021 Jun 4;16(6):e0252751. doi: 10.1371/journal.pone.0252751 (PMC8177625; doi:10.1371/journal.pone.0252751)
Supplement: S1 Fig — a: Methodological quality graph: authors’ judgment about each methodological quality item presented as percentages across all included studies; b: Methodological quality summary: authors’ judgment about each methodological quality item for each included study, “+” low risk of bias; “?” unclear risk of bias; “-” high risk of bias. (DOC) [file pone.0252751.s001.doc]

**
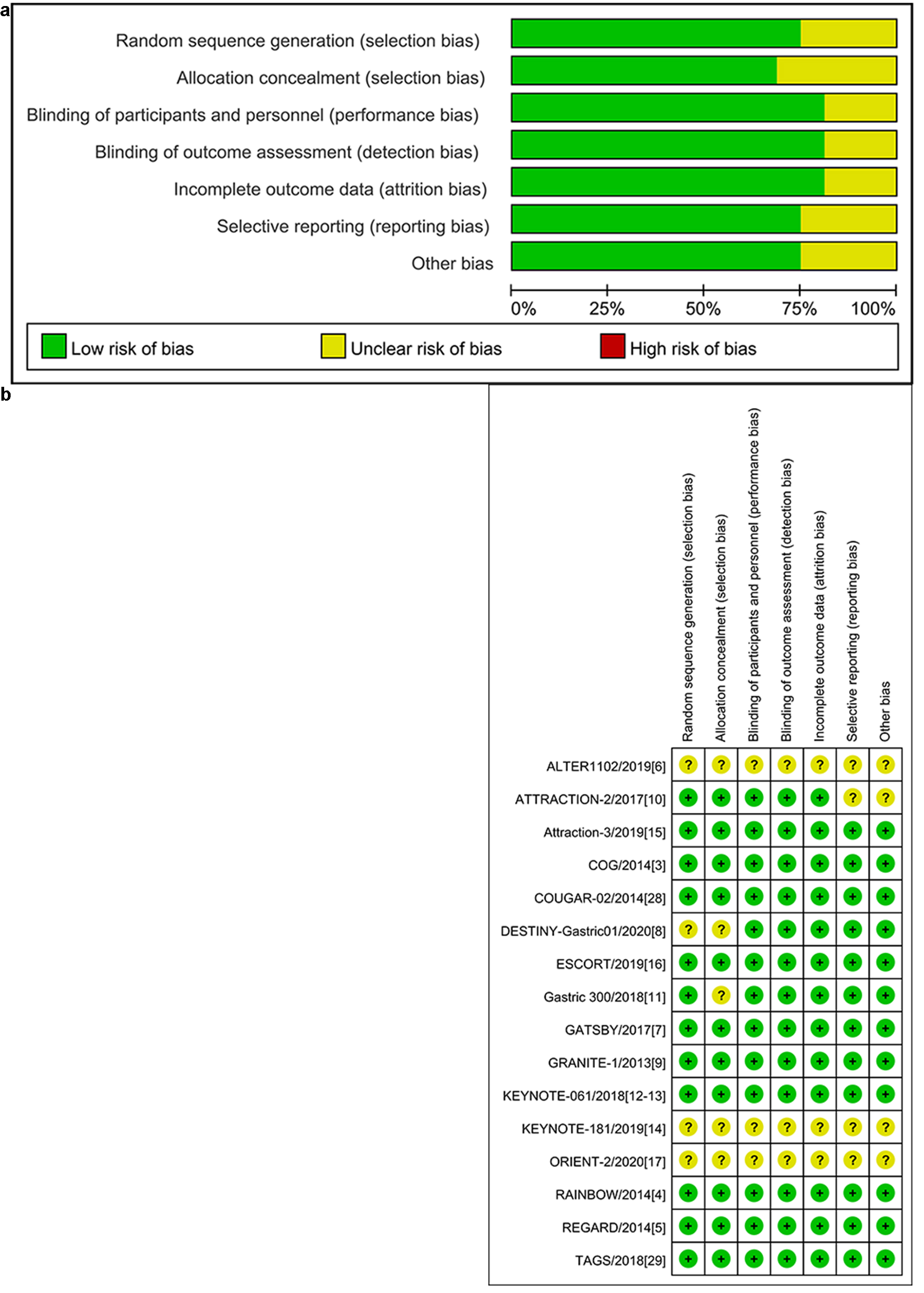
**

**S1 Fig** Assessment of risk of bias. a: Methodological quality graph: authors’ judgment about each methodological quality item presented as percentages across all included studies; b: Methodological quality summary: authors’ judgment about each methodological quality item for each included study, “+” low risk of bias; “?” unclear risk of bias; “-” high risk of bias.
