# Supplementary material for: Comparative efficacy of treatments for previously treated patients with advanced esophageal and esophagogastric junction cancer: A network meta-analysis
Source: PLoS One. 2021 Jun 4;16(6):e0252751. doi: 10.1371/journal.pone.0252751 (PMC8177625; doi:10.1371/journal.pone.0252751)
Supplement: S3 Fig — OS, overall survival; PFS, progression-free survival; ORR, objective response rate; SAE, serious adverse event; SUCRA, surface under the cumulative ranking; T-DXd, trastuzumab deruxtecan; CT, chemotherapy; BSC, best supportive care. (DOC) [file pone.0252751.s003.doc]

**
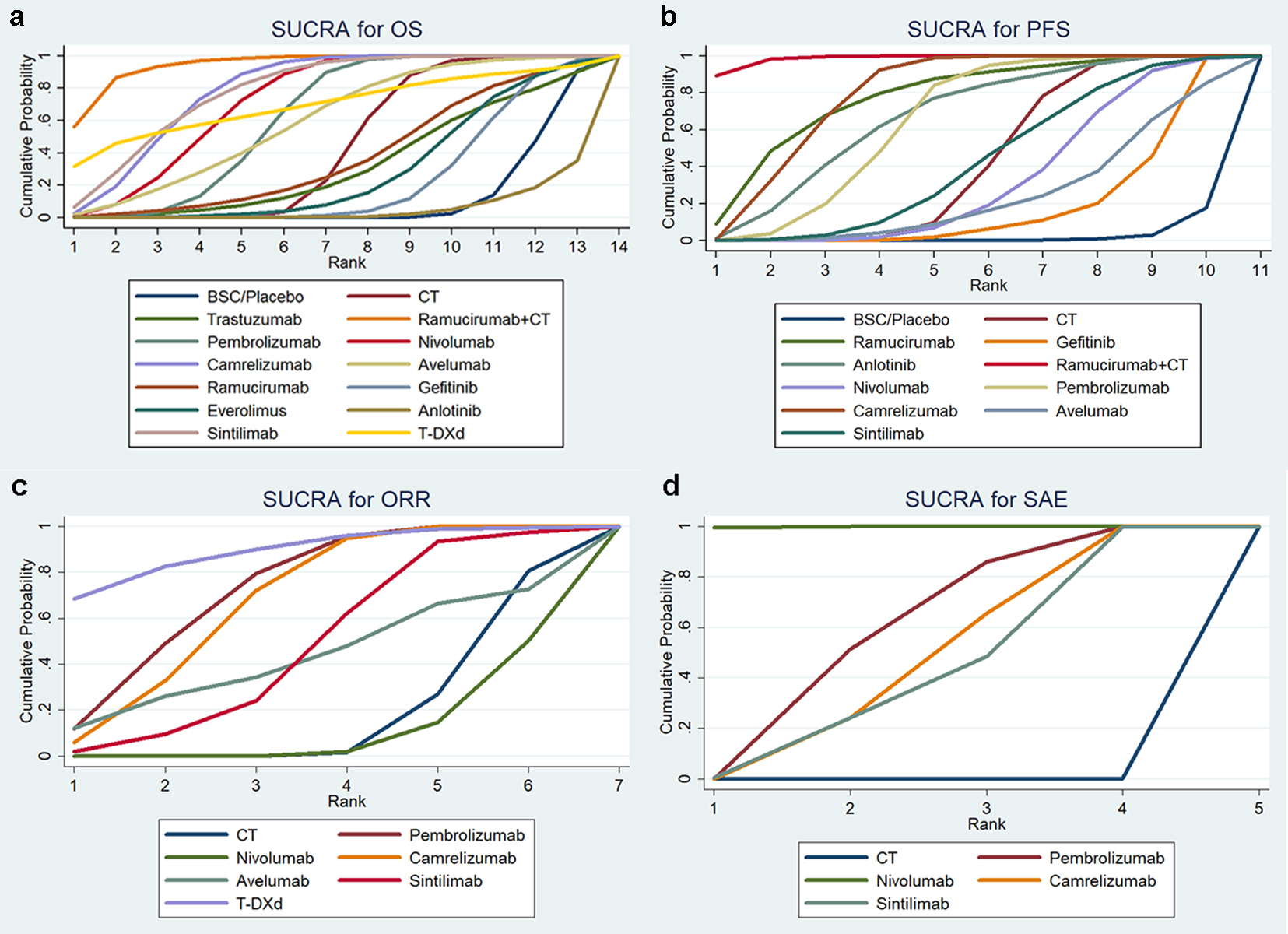
**

**S3 Fig** Treatment ranking curves based on SUCRA. OS, overall survival; PFS, progression-free survival; ORR, objective response rate; SAE, serious adverse event; SUCRA, surface under the cumulative ranking; T-DXd, trastuzumab deruxtecan; CT, chemotherapy; BSC, best supportive care.
