# Supplementary material for: Comparative efficacy of treatments for previously treated patients with advanced esophageal and esophagogastric junction cancer: A network meta-analysis
Source: PLoS One. 2021 Jun 4;16(6):e0252751. doi: 10.1371/journal.pone.0252751 (PMC8177625; doi:10.1371/journal.pone.0252751)
Supplement: S4 Fig — a, sample size; b, median age; c, median follow up time. T-DXd, trastuzumab deruxtecan; CT, chemotherapy; BSC, best supportive care. (DOC) [file pone.0252751.s004.doc]

**
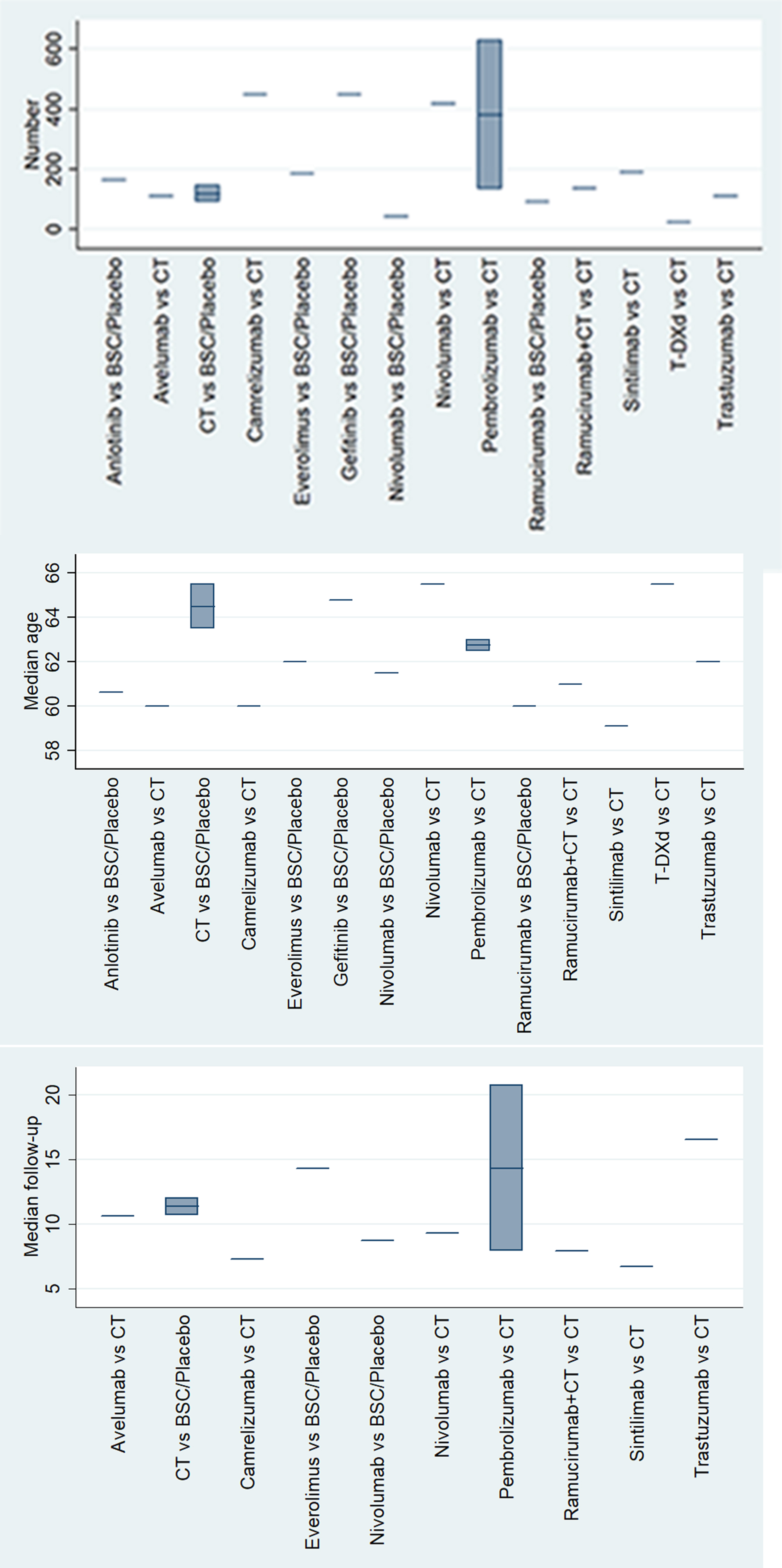
**

**S4 Fig** Assessment of transitivity among included trials. a, sample size; b, median age; c, median follow up time. T-DXd, trastuzumab deruxtecan; CT, chemotherapy; BSC, best supportive care.
