# Supplementary material for: Comparative efficacy of treatments for previously treated patients with advanced esophageal and esophagogastric junction cancer: A network meta-analysis
Source: PLoS One. 2021 Jun 4;16(6):e0252751. doi: 10.1371/journal.pone.0252751 (PMC8177625; doi:10.1371/journal.pone.0252751)
Supplement: S5 Fig — CT, chemotherapy; BSC, best supportive care. (DOC) [file pone.0252751.s005.doc]

**
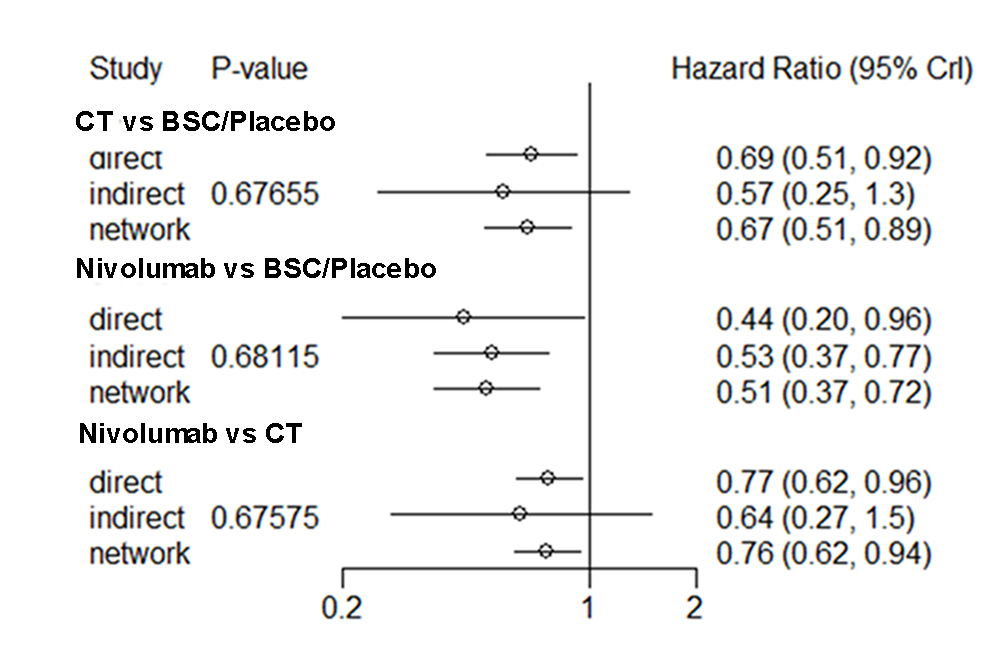
**

**S5 Fig** Inconsistency evaluation by node-splitting analysis for overall survival. CT, chemotherapy; BSC, best supportive care.
