# Supplementary material for: Comparative efficacy of treatments for previously treated patients with advanced esophageal and esophagogastric junction cancer: A network meta-analysis
Source: PLoS One. 2021 Jun 4;16(6):e0252751. doi: 10.1371/journal.pone.0252751 (PMC8177625; doi:10.1371/journal.pone.0252751)
Supplement: S2 Table — (DOC) [file pone.0252751.s007.doc]

**S2 Table** Search strategy

**a:** Search strategy in PubMed

| # | Query |
| --- | --- |
| #1 | “Esophageal Neoplasms”[mh] |
| #2 | Esophageal Neoplasm[tiab] OR Esophagus Neoplasm[tiab] OR Esophagus Cancer[tiab] OR Esophageal Cancer[tiab] OR Esophageal Cancers[tiab] |
| #3 | Esophageal[tiab] OR Oesophageal[tiab] OR Esophagus[tiab] OR Esophag*[tiab] OR Oesophag*[tiab] |
| #4 | Cancer*[tiab] OR Tumour*[tiab] OR Tumor[tiab] OR Neoplasm*[tiab] OR Carcinoma[tiab] |
| #5 | #3 AND #4 |
| #6 | #1 OR #2 OR #5 |
| #7 | "Esophagogastric Junction"[Mesh] |
| #8 | Junction, Esophagogastric[tiab] OR Gastroesophageal Junction[tiab] OR Gastroesophageal Junctions[tiab] OR Junction, Gastroesophageal[tiab] OR Junctions, Gastroesophageal[tiab] |
| #9 | #7 OR #8 |
| #10 | #9 AND #4 |
| #11 | #6 OR #10 |
| #12 | Advanced[tiab] OR Stage IV[tiab] OR Stage 4[tiab] OR Stage Four[tiab] OR StageIIIB[tiab] OR Metastatic[tiab] OR Metastases[tiab] |
| #13 | Second-line[tiab] OR Third-line[tiab] OR Refractory[tiab] OR Previously treated[tiab] |
| #14 | Chemoradiotherapy[tiab] OR Chemotherapy[tiab] OR Chemotherap*[tiab] OR Chemoradi*[tiab] OR Radiochemo*[tiab] OR Radiation therapy[tiab] OR Radiotherapy[tiab] |
| #15 | EGFR[tiab] OR Epidermal Growth Factor Receptor[tiab] OR Cetuximab[tiab] OR Panitumumab[tiab] OR Matuzumab[tiab] OR Nimotuzumab[tiab] OR Tyrosine Kinase Inhibitor[tiab] OR TKI[tiab] OR Gefitinib[tiab] OR Erlotinib[tiab] |
| #16 | Vascular Endothelial Growth Factor[tiab] OR VEGF[tiab] OR VEGFR[tiab] OR Bevacizumab[tiab] OR Sunitinib[tiab] OR Ramucirumab[tiab] OR Apatinib[Title/Abstract] OR Sorafenib[tiab] OR Apatinib[tiab] OR Regorafenib[tiab] OR Anlotinib[tiab] |
| #17 | HER2[tiab] OR HER-2[tiab] OR Trastuzumab[tiab] OR Pertuzumab[tiab] OR Lapatinib[tiab] |
| #18 | c-Met[tiab] OR Onartuzumab[tiab] OR Rilotumumab[tiab] |
| #19 | Programmed Death Ligand 1[tiab] OR PD-L1[tiab] OR Programmed Death 1[tiab] OR PD-1[tiab] OR Anti-Programmed Death Ligand 1[tiab] OR Anti-PD-L1[tiab] OR Anti-Programmed Death 1[tiab] OR Anti-PD-1[tiab] OR Atezolizumab[tiab] OR Durvalumab[tiab] OR Nivolumab[tiab] OR Pembrolizumab[tiab] OR Avelumab[tiab] OR Camrelizumab[tiab] OR Anti-Cytotoxic T-lymphocyte antigen 4[tiab] OR Anti-CTLA-4[tiab] OR Ipilimumab[tiab] OR Tremelimumab[tiab] OR Immunotherapy[tiab] OR Immune checkpoint inhibitors[tiab] OR ICI[tiab] |
| #20 | #14 OR #15 OR #16 OR #17 OR #18 OR #19 |
| #21 | Randomized Controlled Tial[pt] |
| #22 | Controlled Cinical Trial[pt] |
| #23 | Randomized[tiab] |
| #24 | Placebo[tiab] |
| #25 | Randomly[tiab] |
| #26  #27 | Trial[tiab]  Drug Therapy[sh] |
| #28 | Groups[tiab] |
| #29 | #21 OR #22 OR #23 OR #24 OR #25 OR #26 OR #27 OR #28 |
| #30 | Animals[mh] |
| #31 | Humans[mh] |
| #32 | #30 NOT #31 |
| #33 | #29 NOT #32 |
| #34 | #11 AND #12 AND #13 AND #20 AND #33 |

**b:** Search strategy in Embase

| # | Query |
| --- | --- |
| #1 | ‘esophagus tumor’/exp |
| #2 | ‘esophagus tumor’:ab,ti OR ‘esophageal neoplasm’:ab,ti OR ‘esophagus neoplasm’:ab,ti OR ‘esophagus cancer’:ab,ti OR ‘esophageal cancer’:ab,ti OR ‘esophageal cancers’:ab,ti OR ‘gastro-esophageal junction neoplasms’:ab,ti OR ‘esophagogastric junction neoplasms’:ab,ti |
| #3 | ‘esophageal’:ab,ti OR ‘oesophageal’:ab,ti OR ‘esophagus’:ab,ti OR ‘esophag*’:ab,ti OR ‘oesophag*’:ab,ti |
| #4 | ‘cancer*’:ab,ti OR ‘tumour*’:ab,ti OR ‘tumor’:ab,ti OR ‘neoplasm*’:ab,ti OR ‘carcinoma’:ab,ti |
| #5 | #3 AND #4 |
| #6 | #1 OR #2 OR #5 |
| #7 | ‘gastroesophageal junction’/exp |
| #8 | ‘junction, esophagogastric’:ab,ti OR ‘gastroesophageal junction’:ab,ti OR ‘gastroesophageal Junctions’:ab,ti OR ‘junction, gastroesophageal’:ab,ti OR ‘junctions, gastroesophageal’:ab,ti |
| #9 | #7 OR #8 |
| #10 | #9 AND #4 |
| #11 | #6 OR #10 |
| #12 | ‘advanced’:ab,ti OR ‘stage IV’:ab,ti OR ‘stage 4’:ab,ti OR ‘stage four’:ab,ti OR ‘stageIIIB’:ab,ti OR ‘metastatic’:ab,ti OR ‘metastases’:ab,ti |
| #13 | ‘second-line’:ab,ti OR ‘third-line’:ab,ti OR ‘refractory’:ab,ti OR ‘previously treated’:ab,ti |
| #14 | ‘chemoradiotherapy’:ab,ti OR ‘chemotherapy’:ab,ti OR ‘chemotherap*’:ab,ti OR ‘chemoradi*’:ab,ti OR ‘radiochemo*’:ab,ti OR ‘radiation therapy’:ab,ti OR ‘radiotherapy’:ab,ti |
| #15 | ‘EGFR’:ab,ti OR ‘epidermal growth factor receptor’:ab,ti OR ‘cetuximab’:ab,ti OR ‘panitumumab’:ab,ti OR ‘matuzumab’:ab,ti OR ‘nimotuzumab’:ab,ti OR ‘tyrosine kinase inhibitor’:ab,ti OR ‘TKI’:ab,ti OR ‘gefitinib’:ab,ti OR ‘erlotinib’:ab,ti |
| #16 | ‘vascular endothelial growth factor’:ab,ti OR ‘VEGF’:ab,ti OR ‘VEGFR’:ab,ti OR ‘bevacizumab’:ab,ti OR ‘sunitinib’:ab,ti OR ‘ramucirumab’:ab,ti OR ‘apatinib’:ab,ti OR ‘sorafenib’:ab,ti OR ‘apatinib’:ab,ti OR ‘regorafenib’:ab,ti OR ‘anlotinib’:ab,ti |
| #17 | ‘HER2’:ab,ti OR ‘HER-2’:ab,ti OR ‘trastuzumab’:ab,ti OR ‘pertuzumab’:ab,ti OR ‘lapatinib’:ab,ti |
| #18 | ‘c-Met’:ab,ti OR ‘onartuzumab’:ab,ti OR ‘rilotumumab’:ab,ti |
| #19 | ‘programmed death ligand 1’:ab,ti OR ‘PD-L1’:ab,ti OR ‘programmed death 1’:ab,ti OR ‘PD-1’:ab,ti OR ‘anti-programmed death ligand 1’:ab,ti OR ‘anti-PD-L1’:ab,ti OR ‘anti-programmed death 1’:ab,ti OR ‘anti-PD-1’:ab,ti OR ‘atezolizumab’:ab,ti OR ‘durvalumab’:ab,ti OR ‘nivolumab’:ab,ti OR ‘pembrolizumab’:ab,ti OR ‘avelumab’:ab,ti OR ‘camrelizumab’:ab,ti OR ‘anti-cytotoxic T-lymphocyte antigen 4’:ab,ti OR ‘anti-CTLA-4’:ab,ti OR ‘ipilimumab’:ab,ti OR ‘tremelimumab’:ab,ti OR ‘immunotherapy’:ab,ti OR ‘immune checkpoint inhibitors’:ab,ti OR ‘ICI’:ab,ti |
| #20 | #14 OR #15 OR #16 OR #17 OR #18 OR #19 |
| #21 | 'trial':ab,ti |
| #21 | 'random*':ab,ti |
| #22 | 'clinical trial'/de OR 'controlled clinical trial'/de OR 'randomized controlled trial'/de |
| #23 | #21 OR #21 OR #22 |
| #24 | #11 AND #12 AND #13 AND #20 AND #23 |

**c:** Search strategy in Cochrane Library

| # | Query |
| --- | --- |
| #1 | MeSH descriptor: [Esophageal Neoplasms] explode all trees |
| #2 | (esophageal neoplasm OR esophagus neoplasm OR esophagus cancer OR esophageal cancer OR esophageal cancers) |
| #3 | ((esophageal OR oesophageal OR esophagus OR esophag* OR oesophag*) AND (neoplas* OR cancer* OR carcinoma* OR tumour* or tumor)) |
| #4 | MeSH descriptor: [Esophagogastric Junction] explode all trees |
| #5 | (junction, esophagogastric OR gastroesophageal junction OR gastroesophageal junctions OR junction, gastroesophageal OR junctions, gastroesophageal) |
| #6 | (neoplas* OR cancer* OR carcinoma* OR tumour* or tumor) |
| #7 | #4 OR #5 |
| #8 | #7 AND #6 |
| #9 | #1 OR #2 OR #3 OR #8 |
| #10 | (advanced OR stage IV OR stage 4 OR stage four OR stageIIIB OR metastatic OR metastases) |
| #11 | (second-line OR third-line OR refractory OR previously treated) |
| #12 | (chemoradiotherapy OR chemotherapy OR chemotherap* OR chemoradi* OR radiochemo* OR radiation therapy OR radiotherapy) |
| #13 | (EGFR OR epidermal growth factor receptor OR cetuximab OR panitumumab OR matuzumab OR nimotuzumab OR tyrosine kinase inhibitor OR TKI OR gefitinib OR erlotinib) |
| #14 | (vascular endothelial growth factor OR VEGF OR VEGFR OR bevacizumab OR sunitinib OR ramucirumab OR apatinib OR sorafenib OR apatinib OR regorafenib OR anlotinib) |
| #15 | (HER2 OR HER-2 OR Trastuzumab OR Pertuzumab OR Lapatinib) |
| #16 | (c-Met OR Onartuzumab OR Rilotumumab) |
| #17 | (Programmed Death Ligand 1 OR PD-L1 OR Programmed Death 1 OR PD-1 OR Anti-Programmed Death Ligand 1 OR Anti-PD-L1 OR Anti-Programmed Death 1 OR Anti-PD-1 OR Atezolizumab OR Durvalumab OR Nivolumab OR Pembrolizumab OR Avelumab OR Camrelizumab OR Anti-Cytotoxic T-lymphocyte antigen 4 OR Anti-CTLA-4 OR Ipilimumab OR Tremelimumab OR Immunotherapy OR Immune checkpoint inhibitors OR ICI) |
| #18 | #12 OR #13 OR #14 OR #15 OR #16 OR #17 |
| #19 | #9 AND #10 AND #11 AND #18 |

**d:** Search strategy in Web of Science

| # | Query |
| --- | --- |
| #1 | TS=("esophageal neoplasm” OR “esophagus neoplasm” OR “esophagus cancer” OR “esophageal cancer” OR “esophageal cancers” OR ((esophageal OR oesophageal OR esophagus OR esophag* OR oesophag*) AND (neoplas* OR cancer* OR carcinoma* OR tumour* or tumor))) |
| #2 | TS=(("esophagogastric junction” OR “junction, esophagogastric” OR “gastroesophageal junction” OR “gastroesophageal junctions” OR “junction, gastroesophageal” OR “junctions, jastroesophageal”) AND (neoplas* OR cancer* OR carcinoma* OR tumour* or tumor)) |
| #3 | #1 OR #2 |
| #4 | TS=(“advanced” OR “stage IV” OR “stage 4” OR “stage Four” OR “stageIIIB” OR “metastatic” OR “metastases”) |
| #5 | TS=(“second-line” OR “third-line” OR “refractory” OR “previously treated”) |
| #6 | TS=(“chemoradiotherapy” OR “chemotherapy” OR “chemotherap*” OR “chemoradi*” OR “radiochemo*” OR “radiation therapy” OR “radiotherapy”) |
| #7 | TS=(“EGFR” OR “epidermal growth factor receptor” OR “cetuximab” OR “panitumumab” OR “matuzumab” OR “nimotuzumab” OR “tyrosine kinase inhibitor” OR “TKI” OR “gefitinib” OR “erlotinib”) |
| #8 | TS=(“vascular endothelial growth factor” OR “VEGF” OR “VEGFR” OR “bevacizumab” OR “sunitinib” OR “ramucirumab” OR “apatinib” OR “sorafenib” OR “apatinib” OR “regorafenib” OR “anlotinib”) |
| #9 | TS=(“HER2” OR “HER-2” OR “trastuzumab” OR “pertuzumab” OR “lapatinib”) |
| #10 | TS=(“c-Met” OR “onartuzumab” OR “rilotumumab”) |
| #11 | TS=(“programmed death ligand 1” OR “PD-L1” OR “programmed death 1” OR “PD-1” OR “anti-programmed death ligand 1” OR “anti-PD-L1” OR “anti-programmed death 1” OR “anti-PD-1” OR “atezolizumab” OR “durvalumab” OR “nivolumab” OR “pembrolizumab” OR “avelumab” OR “camrelizumab” OR “anti-cytotoxic T-lymphocyte antigen 4” OR “anti-CTLA-4” OR “ipilimumab” OR “tremelimumab” OR “immunotherapy” OR “immune checkpoint inhibitors” OR “ICI”) |
| #12 | #6 OR #7 OR #8 OR #9 OR #10 OR #11 |
| #13 | TS=("randomized controlled trial" OR "controlled clinical trial" OR "clinical trial" OR "random*" OR "rct*" OR "crossover" OR "masked” OR “blind*" OR "placebo*") |
| #14 | #3 AND #4 AND #5 AND #12 AND #13 |
