# Supplementary material for: Comparative efficacy of treatments for previously treated patients with advanced esophageal and esophagogastric junction cancer: A network meta-analysis
Source: PLoS One. 2021 Jun 4;16(6):e0252751. doi: 10.1371/journal.pone.0252751 (PMC8177625; doi:10.1371/journal.pone.0252751)
Supplement: S3 Table — (DOC) [file pone.0252751.s008.doc]

**S3 Table** Comparisons of the fit of consistency and inconsistency models

|  | Overall | | | | Subgroup | | | |
| --- | --- | --- | --- | --- | --- | --- | --- | --- |
| Model | OS | PFS | ORR | SAEs | OS (AC) | OS (SCC) | PFS (AC) | PFS (SCC) |
| Consistency | 29.30 | 19.93 | 25.03 | 16.05 | 18.69 | 7.98 | 10.03 | 8.02 |
| Inconsistency | 31.29 | 19.93 | 25.02 | 16.08 | 18.74 | 7.99 | 9.98 | 7.99 |

Abbreviations: OS, overall survival; PFS, progression-free survival; ORR, objective response rate; SAEs, serious adverse events; AC, adenocarcinoma; SCC, squamous cell carcinoma
