# Supplementary material for: Comparative efficacy of treatments for previously treated patients with advanced esophageal and esophagogastric junction cancer: A network meta-analysis
Source: PLoS One. 2021 Jun 4;16(6):e0252751. doi: 10.1371/journal.pone.0252751 (PMC8177625; doi:10.1371/journal.pone.0252751)
Supplement: S4 Table — (DOC) [file pone.0252751.s009.doc]

**S4 Table** Results of sensitivity analysis

| **a. Omitting trials with sample size less than 50** | | | | | | | | | | | | |
| --- | --- | --- | --- | --- | --- | --- | --- | --- | --- | --- | --- | --- |
| HR with 95%CI for OS | | | | | | | | | | | | |
| Ramucirumab+CT |  |  |  |  |  |  |  |  |  |  |  |  |
| 0.73(0.46-1.15) | Camrelizumab |  |  |  |  |  |  |  |  |  |  |  |
| 0.74(0.44-1.24) | 1.02(0.69-1.51) | Sintilimab |  |  |  |  |  |  |  |  |  |  |
| 0.67(0.43-1.07) | 0.92(0.68-1.25) | 0.91(0.61-1.35) | Nivolumab |  |  |  |  |  |  |  |  |  |
| **0.62(0.40-0.95)** | 0.84(0.65-1.09) | 0.83(0.58-1.20) | 0.92(0.70-1.19) | Pembrolizumab |  |  |  |  |  |  |  |  |
| 0.60(0.33-1.55) | 0.83(0.51-1.33) | 0.81(0.47-1.39) | 0.90(0.55-1.45) | 0.98(0.62-1.55) | Avelumab |  |  |  |  |  |  |  |
| **0.52(0.35-0.78)** | **0.71(0.58-0.88)** | **0.70(0.50-0.97)** | **0.77(0.62-0.96)** | **0.84(0.72-0.98)** | 0.86(0.56-1.33) | CT |  |  |  |  |  |  |
| **0.47(0.24-0.93)** | 0.64(0.35-1.17) | 0.63(0.33-1.21) | 0.70(0.38-1.26) | 0.76(0.43-1.36) | 0.78(0.38-1.57) | 0.90(0.52-1.58) | Ramucirumab |  |  |  |  |  |
| **0.44(0.23-0.85)** | 0.60(0.34-1.05) | 0.59(0.32-1.10) | 0.65(0.37-1.15) | 0.71(0.41-1.23) | 0.73(0.37-1.44) | 0.85(0.50-1.43) | 0.94(0.43-2.02) | Trastuzumab |  |  |  |  |
| **0.42(0.23-0.77)** | **0.58(0.36-0.95)** | 0.57(0.33-1.00) | 0.63(0.39-1.03) | 0.69(0.43-1.10) | 0.71(0.38-1.30) | 0.82(0.53-1.27) | 0.91(0.51-1.61) | 0.97(0.49-1.93) | Everolimus |  |  |  |
| **0.40(0.23-0.68)** | **0.54(0.36-0.82)** | **0.53(0.33-0.87)** | **0.59(0.39-0.89)** | **0.64(0.44-0.94)** | 0.66(0.37-1.15) | 0.77(0.54-1.08) | 0.85(0.51-1.41) | 0.90(0.48-1.71) | 0.93(0.64-1.36) | Gefitinib |  |  |
| **0.36(0.22-0.59)** | **0.49(0.34-0.70)** | **0.48(0.31-0.75)** | **0.53(0.37-0.76)** | **0.58(0.42-0.81)** | 0.59(0.35-1.00) | **0.69(0.51-0.93)** | 0.76(0.47-1.22) | 0.81(0.44-1.50) | 0.84(0.61-1.16) | 0.90(0.74-1.09) | Placebo/BSC |  |
| **0.30(0.16-0.58)** | **0.41(0.24-0.71)** | **0.41(0.23-0.74)** | **0.45(0.26-0.77)** | **0.49(0.29-0.83)** | **0.50(0.26-0.98)** | **0.58(0.36-0.96)** | 0.65(0.35-1.20) | 0.75(0.26-2.18) | 0.71(0.43-1.19) | 0.76(0.49-1.19) | 0.85(0.57-1.26) | Anlotinib |
| **b. Omitting phase II and phase II/III trials** | | | | | | | | | | | | |
| HR with 95%CI for OS | | | | | | | | | | | | |
| Ramucirumab+CT |  |  |  |  |  |  |  |  |  |  |  |  |
| 0.73(0.47-1.16) | Camrelizumab |  |  |  |  |  |  |  |  |  |  |  |
| 0.68(0.44-1.08) | 0.93(0.69-1.26) | Nivolumab |  |  |  |  |  |  |  |  |  |  |
| **0.62(0.40-0.95)** | 0.84(0.65-1.09) | 0.90(0.69-1.17) | Pembrolizumab |  |  |  |  |  |  |  |  |  |
| 0.60(0.34-1.08) | 0.82(0.51-1.33) | 0.88(0.54-1.43) | 0.98(0.62-1.54) | Avelumab |  |  |  |  |  |  |  |  |
| **0.52(0.35-0.78)** | **0.71(0.57-0.88)** | **0.76(0.62-0.94)** | **0.84(0.72-0.98)** | 0.86(0.56-1.33) | CT |  |  |  |  |  |  |  |
| **0.46(0.23-0.91)** | 0.63(0.35-1.13) | 0.67(0.38-1.21) | 0.75(0.42-1.32) | 0.76(0.38-1.54) | 0.89(0.51-1.54) | Ramucirumab |  |  |  |  |  |  |
| **0.42(0.23-0.75)** | **0.57(0.35-0.92)** | **0.61(0.38-0.97)** | 0.68(0.43-1.06) | 0.69(0.38-1.26) | 0.80(0.52-1.23) | 0.91(0.51-1.60) | Everolimus |  |  |  |  |  |
| **0.39(0.23-0.66)** | **0.53(0.36-0.79)** | **0.57(0.39-0.84)** | **0.63(0.44-0.91)** | 0.65(0.37-1.12) | 0.75(0.53-1.05) | 0.85(0.50-1.40) | 0.94(0.64-1.36) | Gefitinib |  |  |  |  |
| **0.35(0.21-0.57)** | **0.48(0.34-0.68)** | **0.51(0.37-0.72)** | **0.57(0.41-0.78)** | **0.58(0.35-0.97)** | **0.67(0.51-0.89)** | 0.76(0.47-1.22) | 0.84(0.61-1.16) | 0.90(0.74-1.09) | Placebo/BSC |  |  |  |

Abbreviations: OS, overall survival; HR, hazard ratios; CI, confidence interval; CT, chemotherapy; BSC, best supportive care.
