# Supplementary material for: Comparative efficacy of treatments for previously treated patients with advanced esophageal and esophagogastric junction cancer: A network meta-analysis
Source: PLoS One. 2021 Jun 4;16(6):e0252751. doi: 10.1371/journal.pone.0252751 (PMC8177625; doi:10.1371/journal.pone.0252751)
Supplement: Attachment — Submitted filename: reviewer.docx [file pone.0252751.s010.docx]

Major revision:

1. There was no quality assessment of the included studies;

2. The charts of sensitivity analysis and heterogeneity analysis were not provided.
